# Identical versus compatible blood typing: investigating best practices in lung transplantation

**DOI:** 10.3389/ti.2026.15722

**Published:** 2026-05-22

**Authors:** Helena Bugacov, Cristina Cusmai, Kenneth Li, Anne Michelle Montal, Gbalekan Dawodu, Tian Sun, Stephanie Tuminello, Shubham Gulati, Kuo-Chang Tseng, Joshua Taylor Bernstein, Daniel Laskey, Harish Seethamraju, Scott Scheinin

**Affiliations:** 1 Department of Thoracic Surgery, Icahn School of Medicine at Mount Sinai, New York, NY, United States; 2 University of California Los Angeles Eli and Edythe Broad Center of Regenerative Medicine and Stem Cell Research, Los Angeles, CA, United States; 3 Stevens Institute of Technology School of Business, Hoboken, United States

**Keywords:** ABO blood group system, ABO blood type, lung, lung transplant, allocation

## Abstract

This study investigated acute rejection in ABO-compatible versus ABO-identical lung transplants from 2005–2023, with a secondary objective of comparing 5-year survival. Lung transplantation improves quality of life and survival; however, organ scarcity remains a major challenge. While ABO-identical matching has traditionally been preferred, evolving allocation policies and research have increased the use of ABO-compatible transplants to expand the donor pool. A retrospective cohort study using the UNOS database included adult recipients (n = 32,761). Comparisons were made between ABO-identical (n = 30,347) and ABO-compatible (n = 2,414) groups. Logistic regression assessed acute rejection, and Kaplan-Meier and Cox proportional hazards models evaluated 5-year survival. There was no significant difference in acute rejection (p = 0.99; OR = 1.03, 95% CI 0.86–1.21). ABO-identical transplants showed improved 5-year survival (p = 0.019; HR = 0.91, 95% CI 0.85–0.98), with benefits limited to recipients with obstructive lung disease (p = 0.043) and those in the highest LAS quartile (p = 0.014). The differential benefit of this modest association between ABO-identical matching and survival in this subpopulation remains unclear. Overall, ABO-compatible transplantation safely expands donor availability without increasing rejection risk.

## Graphical Abstract

**Figure:**
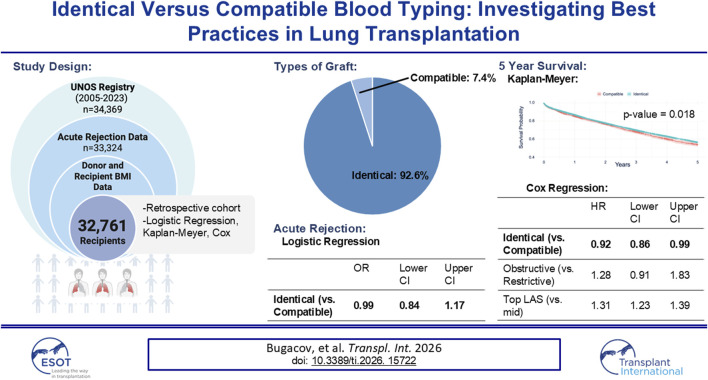

## Introduction

Lung transplantation offers improved quality of life and survival, with approximately 88.4% 1-year survival rates in 2018–2023 [1]. Globally, the incidence of lung transplant surpasses more than 4,600 cases annually, the majority being bilateral, with a median survival of 6.2 years in the adult population [2]. In 2022, 2,743 lung transplants were performed, a number that has been gradually increasing since the pandemic, alongside the addition of 3,161 candidates to the waiting list [3]. However, organ scarcity remains a significant challenge [3]. Donor-recipient matching is an evolving strategy aimed at optimizing the best organ for the most medically urgent recipient [3]. Prior to 2023, lung allocation was determined by a classification-based system [4]. Candidates were initially arranged into ordered groups based on blood type identical and within 250 miles of the donor hospital [5]. Within each group, the candidates were then ranked preferentially by Lung Allocation Scores (LAS) [5].

In early 2023, the lung allocation policy converted to a continuous distribution framework, which utilizes a composite allocation score (CAS) to determine the preferential order of candidates when a medically suitable donor becomes available [5]. This change was implemented to improve fairness, decrease waitlist mortality, increase access to lung transplants for the most medically urgent candidates, and improve patient outcomes in lung transplant allocation [5]. The Lung CAS includes nine candidate attributes, which include two components of the LAS. These are expected 1-year waiting list mortality without a transplant (WLAUC), expected 5-year post-transplant survival, blood type matching, CPRA (HLA antibody sensitization), height, pediatric status, prior living donor status, travel efficiency (including travel and transportation costs), and proximity efficiency [5]. Blood type matching (ABO-compatible, see Methods “ABO Matching”) allows recipients with harder-to-match blood types to have increased priority for potential donors compared to those with more common blood types, providing a recipient with more prospective donors, compared to blood type identical matching [5].

Traditionally, ABO-identical matching has been preferred in lung transplantation due to the perceived lower risk of rejection [6]. Non-identical ABO or ABO-compatible lung transplantation is an area of ongoing research to expand the donor pool for recipients, with prior studies suggesting good outcomes with ABO compatible transplants [6–9]. These studies were, however, limited due to sample size and single-center studies, for instance, investigating 325 patients between 1990 and 2016 [9]. Taghavi et al. examined 6,655 adult double lung transplantations from May 2005 to December 2011, and their multivariate analysis demonstrated no association with long-term or short-term mortality [8]. There was no clarity, however, on whether acute and chronic rejection were more common among ABO compatible organs.

Our study builds upon previous research by comparing outcomes between recipients of identical and compatible ABO lung transplants, focusing on patient survival and acute rejection rates. Acute rejection is a clinically meaningful marker of immune injury due to the possibility of ABO-incompatibility triggering acute or hyperacute antibody-mediated graft rejection [10] and complications such as passenger lymphocyte syndrome [11], as well as it being a risk factor associated with the development of chronic lung allograft dysfunction [12]. 5-year survival is a robustly captured endpoint in UNOS that has notably been incorporated in the lung allocation policy to prioritize candidates, reflecting consensus that this endpoint is an important measure of transplant benefit [13]. The aim of our study is to gain insight into the role of ABO compatibility in optimizing lung transplant outcomes and inform clinical decision-making to enhance donor organ utilization.

## Materials and Methods

### Study design and population

United Network for Organ Sharing (UNOS) is a non-profit organization contracted by the federal government to manage organ donation and allocation. The Standard Transplant Analysis and Research data files, a public-access dataset, were obtained from the UNOS registry. All lung transplants, both single and double, performed in the United States from June 2005 to June 2023 were examined. ABO compatible transplants were compared to ABO identical transplants, with an endpoint of risk-adjusted all-cause mortality. Acute rejection, which was defined as any recorded acute rejection episode in the follow-up window of our dataset, was used as a secondary endpoint.

### Study population (inclusion/exclusion criteria)

We included all de-identified lung transplants in the UNOS registry from June 2005-June 2023 with donor-recipient ABO match categorized as ABO-identical or compatible. We excluded pediatric recipients, recipients without an acute rejection episode status classification, recipients without a recipient BMI or associated donor BMI, and cases without ischemic time. Re-transplantations and multi-organ transplants were not excluded. For survival analyses, recipients missing survival time were excluded and follow-up was censored at 5 years.

### Primary exposure

The ABO blood group compatibility was classified into “Identical” and “Compatible.”

### Primary outcome

The primary outcome used in our analysis was risk-adjusted all-cause mortality.

### Confounding variables

Additional variables examined included recipient age, donor age, lung transplant type (single or double), recipient gender, and medical conditions of the recipient before transplantation, categorized as “hospitalized but not ICU”, “Intensive Care”, or “Not Hospitalized”. We also maintained the four major categories from LAS in this analysis, including obstructive lung disease, pulmonary hypertension, cystic fibrosis, and restrictive lung disease [14]. We also used the UNOS data set to calculate age difference as the absolute age difference between the recipient and donor. We calculated BMI differences as the absolute difference in BMI between the recipient and donor, and height differences as the absolute height difference between the recipient and donor. The initial Lung Allocation Score was cut at the 25th (33.74) and 75th (44.96) percentiles to define three groups (below 1st, 1st–4th, above 4th).

### Statistical analysis

Univariate comparisons between groups based on ABO compatibility and acute rejection event used chi-square tests for categorical variables and t-tests for continuous variables. Statistical significance was defined as a two-tailed p-value of less than 0.05. Multivariable logistic regression was utilized to assess the association between ABO status and acute rejection ABO status and ICU status, ICU status and blood type, and high vs. low LAS score as a function of blood type.

Kaplan-Meier survival analysis was used to estimate overall survival probabilities over 5 years post-transplant, capped at 1825 days (5 Years), and the status of each patient (alive or deceased). A Cox proportional hazards model was used to assess the impact of ABO status on survival, adjusting for age, allocation quartile, and disease category, transplant type, and gender.

Data organization, analysis, manipulation, visualization, and statistical analysis were performed using R statistical software while running and utilizing the following packages: survival, survminer, dplyr, ggplot2, tidyverse, readxl, and compareGroups [14–19].

### Ethics statement

According to our institution’s IRB office this study does not meet the criteria for IRB approval as this study does not include identifiable human subjects. This study only contains de-identified UNOS data and is not interacting with any participants or obtaining identifiable data from other sources.

### ABO matching

ABO blood grouping, also known as blood type, is the presence or absence of antigens, labeled A and B, on the surface of a red blood cell (RBC) [20]. In the absence of one or both antigens, antibodies against the antigen are formed [21]. It is these antibodies that cause mismatch reactions. People whose red blood cells display only the A antigen generate serum anti-B antibodies and are labeled as type A^21^. Conversely, those with B-antigen have anti-A-antibodies and are considered type B. Individuals with both A and B antigens do not produce any antibodies and are labeled AB. Lastly, people expressing neither antigen are considered O-type and generate antibodies to both A- and B-type antigens [21].

ABO matching has been a significant cornerstone of transplantation [22]. Type O recipients can only receive blood from donors who are also Type O, as they have circulating antibodies to both A and B antigens [22]. Type A can receive organs from type A or O donors and Type B can receive organs from type B or O donors [22]. Type AB individuals do not have antibodies; therefore, they can receive donors from all blood types. When a recipient and donor are matched to the same ABO type (e.g., a Type A recipient receiving a Type A donor), this is referred to as ABO-identical matching. Minor ABO-incompatibility, or ABO-compatible transplantation, occurs with a blood type O donor lung transplanted into A, B, or AB recipients, or a blood type A and B donor lung transplanted into a AB recipient [22].

Major ABO-incompatibility occurs when an organ with A or B antigens is transplanted into a recipient with corresponding antibodies, most seen when blood type O patients receive lungs from A, B, or AB donors. ABO-incompatible cases have been done only inadvertently due to clerical errors [7, 23, 24].

## Results

**FIGURE 1:**
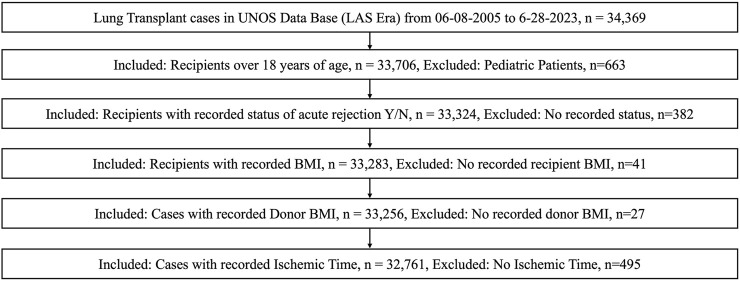
Schematic representing cohort selection.

During the study period, 34,369 patients underwent lung transplantation, as reported by the UNOS database. Pediatric patients under the age of 18 years were excluded, leaving 33,706 adult patients. These were screened for documentation of acute rejection episodes (33,324). Patients with missing data for body mass index (BMI) from either the donor or the recipient were also excluded, as were patients with no recorded ischemic time, resulting in a final retrospective cohort of n = 32,761 (Figure 1). Of this, 30,347 transplants occurred with identical ABO matching, and 2,414 occurred with compatible matching.

### Acute rejection

In the univariate analysis (Table 1), there was no significant difference in gender or acute rejection between identical ABO and compatible matching. The ABO compatible group had an older mean age (58.4 in compatible vs. 57.1 in identical) (age, p < 0.001) and a similar BMI (25.3 in compatible and 25.5 identical) (BMI, p = 0.005). There was a small, but statistically significant difference in the ABO identical group being more likely to be transplanted from the ICU (12.8% in identical vs. 11.8% in compatible) and non-ICU hospitalizations (9.9% in identical vs. 8.0% in compatible). In contrast, the ABO compatible group was more likely to be transplanted from an outpatient setting (80.2% in compatible vs. 77.2% in identical). (Medical conditions, p = 0.002). Patients with low LAS scores are likely to be on the waitlist for a long time as they are not a priority due to their LAS score, which is calculated by their disease severity as a factor. As a result, they are more likely to have compatible ABO matching because they would not otherwise receive a transplant. Additionally, to examine if ABO-compatible grafts were used in more clinically stable patients, we fit a multivariate logistic regression model with ABO-identical vs. ABO-compatible matching as the outcome. We found a modest increase in use of compatible donors in ICU cases vs. non-ICU-cases (OR 1.26, 95% CI 1.04–1.52). We also fit a multivariate logistic regression model with ICU vs. non-ICU status as the outcome, and found more O type patients (who can only receive ABO-identical grafts) in the ICU (OR 1.16, 95% CI 1.07–1.24). Additionally, we fit a multivariate logistic regression model with high LAS vs. non-high LAS as the outcome and found that O type patients were also in the highest allocation quartile (OR 1.09, 95% CI 1.03–1.15).

**TABLE 1:** Summary of patient characteristics (Univariate/Unadjusted analysis).

Variable	Compatible, n = 2,414	Identical, n = 31,235	p-value
Acute Rejection	​	​	0.99
No	2251 (93.2%)	28289 (93.2%)	​
Yes	163 (6.75%)	2058 (6.78%)	​
Recipient Gender	​	​	0.437
F	997 (40.5%)	12031 (39.6%)	​
M	1437 (59.5%)	18316 (60.4%)	​
Recipient Age	58.4 (12.0)	57.1 (12.6)	<0.001
Recipient BMI	25.3 (4.35)	25.5 (4.55)	0.005
Medical Condition	​	​	0.002
Intensive Care	285 (11.8%)	3888 (12.8%)	​
Hospitalized – Not ICU	193 (8.00%)	3016 (9.94%)	​
Not Hospitalized	1936 (80.2%)	23443 (77.2%)	​
Disease Stratification	​	​	<0.001
Cystic Fibrosis	170 (7.04%)	2464 (8.12%)	​
Obstructive	835 (34.6%)	7619 (25.1%)	​
Other	233 (9.65%)	3496 (11.5%)	​
Restrictive	1176 (48.7%)	16768 (55.3%)	​
Lung Transplantation	​	​	<0.001
Single	798 (33.1%)	8419 (27.7%)	​
Double	1612 (66.9%)	21920 (72.3%)	​
Allocation Quartile	​	​	<0.001
Top	496 (20.5%)	7802 (24.0%)	​
Middle	1134 (47.0%)	15276 (50.3%)	​
Bottom	784 (32.5%)	7269 (24.0%)	​

Comparing recipient diagnosis, patients with obstructive disease were more likely to undergo ABO compatible matching (34.6% in compatible vs. 25.1% in identical). Higher use of ABO-compatible donors in patients with obstructive disease likely reflects real-world patterns in center allocation of lungs. Specifically, patients with obstructive disease were more likely to fall into a lower LAS score (65% of obstructive vs. 9.6% and 13.8% of restrictive and other diagnoses, p < 0.001) and be transplanted from an outpatient setting (92.1% of obstructive vs. 73.8% and 69.8% of restrictive and other diagnoses, p < 0.001). Centers may be more willing to accept ABO-compatible organs to expand donor utilization and shorten wait times for this population of patients. In comparison, restrictive (55.3% identical vs. 48.7% compatible), cystic fibrosis (8.12% identical vs. 7.04% compatible), and all other diagnoses (which includes less ubiquitous diagnoses such as Kartagener’s syndrome, common variable immune deficiency, or associated diagnoses such as pulmonary hypertension) (11.5% identical vs. 9.65% compatible) were more likely to undergo ABO identical matching (recipient diagnoses, p < 0.001). 33.1% of recipients with ABO compatible matching underwent single-lung transplantation. In contrast, 27.7% of recipients with ABO identical matching underwent single-lung transplantation (lung transplant type, p < 0.001, Table 1). When stratified by allocation score, the top third and middle tier scores were more likely to receive an ABO identical matching (24.0% and 50.3% in top and middle third) versus compatible (20.5% and 47% in top and middle third). The lower third of allocation scores, however, were more likely to have ABO compatible matching (32.5%) compared to ABO identical (24.0%). (LAS score, p < 0.001). Reviewing episodes of acute rejection, there was no significant difference found between ABO identical matching and ABO compatible matching (acute rejection, p = 0.990).

Based on the univariate results, we adjusted for recipient age, gender, medical condition, and disease type in the logistic regression model (Table 2). Recipients with restrictive disease and the “other” category were at an increased odds of acute rejection compared to the cystic fibrosis phenotype (Odds Ratio [OR] = 1.28, 95% Confidence Interval [CI]: 1.05–1.56), (OR 1.28, 95% CI = 1.05–1.56). Female recipients also had lower odds of acute rejection than their male counterparts (OR = 0.83, CI: 0.76–0.90). There was no significant risk of acute rejection for the recipient, regardless of medical conditions (Hospitalized-Not ICU, Intensive Care, or Not Hospitalized), when adjusting for confounders identified in the univariate analysis (Table 2).

**TABLE 2:** Multivariate/Adjusted analysis. Logistic regression - Relative odds of Acute rejection episode confounding for ABO status using logistic regression recipient age, recipient gender, medical condition and disease category. Identical blood type, Hospitalized not ICU and Cystic fibrosis were reference categories for analysis.

Comparison	Odds Ratio	Lower CI	Upper CI
ABO Identical vs Compatible	0.99	0.84	1.17
Intensive Care Unit (ICU) vs Not Hospitalized	1.25	1.09	1.42
Hospitalized, not ICU vs Not Hospitalized	1.11	0.95	1.28
Obstructive vs Cystic Fibrosis	1.18	0.95	1.46
Restrictive vs Cystic Fibrosis	1.21	0.99	1.48
Other vs Cystic Fibrosis	1.23	1.01	1.51
Top Quartile Allocation Score vs Middle Quartile	1.22	1.09	1.36
Bottom Quartile Allocation Score vs Middle Quartile	0.98	0.86	1.11
Single vs. Double Transplant	0.68	0.62	0.76

When adjusted for age, gender, medical condition, disease category, and lung transplant type, the multivariate analysis showed no significant difference in acute rejection between the ABO identical and compatible matching (Table 2).

### Overall survival

A Cox proportional hazards regression was used to further investigate the impact of covariates on 5-year survival. Five year survival is reported by the ISHLT data base as it best assesses survival immunogenicity; shorter survival, such as 1 year survival often reflects mortality from infection and acute rejection risk [2]. The factors included gender, ABO status, allocation quartile, disease category (grouped into cystic fibrosis, obstructive, restrictive, and other), and transplant type (single vs. double). The model revealed that ABO-identical matches were associated with a decreased risk of death (Hazard Ratio [HR] = 0.91, 95% CI = 0.85–0.98). Additionally, the highest age quartile of the recipient (HR = 1.48, 95% CI = 1.39–1.58) and the highest allocation score quartile (HR = 1.29, 95% CI = 1.20–1.36) were associated with an increased risk of death. Lastly, recipients with disease in the “other” category were associated with increased risk of death (HR 1.37, 95% CI = 1.25–1.49) (Table 3).

**TABLE 3:** Adjusted overall survival investigated using logistic and Cox proportional hazards regression, adjusted for sex, age, allocation quartile, and disease category.

Comparison	Hazards Ratio	Lower .95	Upper .95
ABO Identical vs Compatible	0.92	0.86	0.99
Top LAS vs Middle LAS	1.31	1.23	1.39
Bottom LAS vs Middle LAS	1.07	1.02	1.13
Obstructive vs Cystic Fibrosis	1.04	0.95	1.13
Restrictive vs Cystic Fibrosis	1	0.92	1.08
Other vs Cystic Fibrosis	1.32	1.21	1.45
Single vs. Double Transplant	0.78	0.75	0.81

**FIGURE 2:**
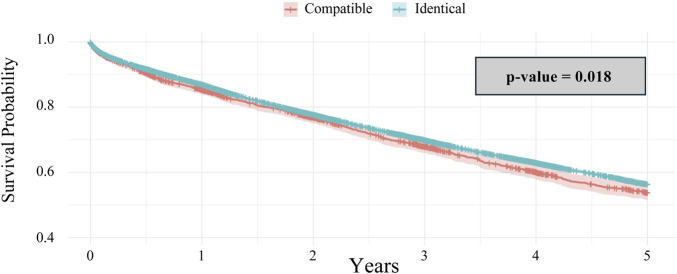
Kaplan-Meier 5-year survival analysis of ABO Compatible vs. Identical Groups.

Across all patients analyzed, the Kaplan-Meier 5-year survival curve demonstrated a significant survival benefit towards ABO-identical matching compared to compatible matching (p = 0.02) (Figure 2).

**FIGURE 3:**
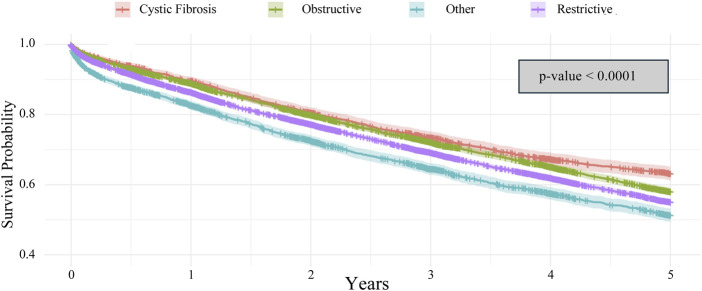
Kaplan-Meier 5-year survival analysis of lung transplant recipients characterized by lung disease.

**FIGURE 4:**
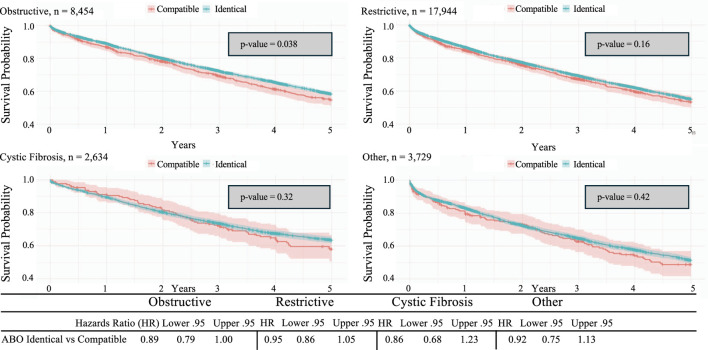
Kaplan-Meier 5-year survival analysis of lung transplant recipients categorized by ABO identical vs. compatible status receiving lung transplantation stratified by lung disease.

A Kaplan-Meier survival analysis was used to compare the 5-year survival for the four different disease categories: cystic fibrosis, obstructive, restrictive, and other. The analysis revealed a significant difference in survival among the different disease groups (p < 0.0001). Cystic fibrosis had a significantly improved 5-year survival, with the other categories having the worst survival outcomes (Figure 3). When assessing stratification between disease processes, only obstructive disease was found to have a significant difference in survival between ABO identical and ABO compatible groups (p = 0.038) (Figure 4). Restrictive, cystic fibrosis, and the other group did not demonstrate a significant survival difference between identical and compatible matching.

**FIGURE 5:**
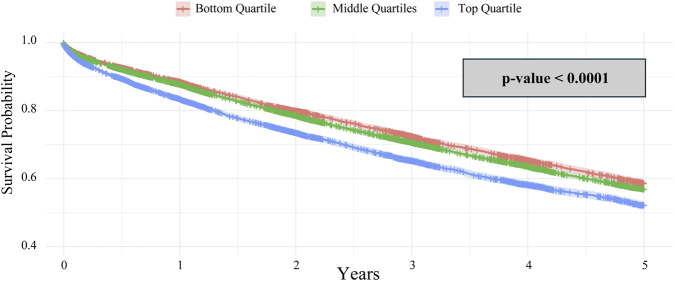
Kaplan-Meier 5-year survival analysis of lung transplant recipients categorized by lung allocation score quartile.

**FIGURE 6:**
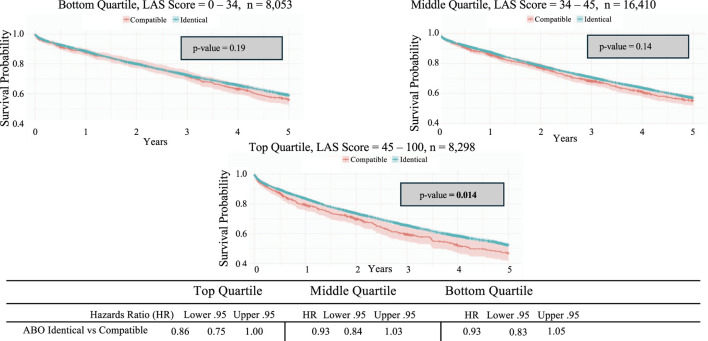
Kaplan-Meier 5-year survival analysis of lung transplant recipients categorized by lung allocation score quartile and stratified by ABO identical vs. compatible status.

Another Kaplan-Meier survival analysis compared the 5-year survival rates of the three allocation score categories: below 1st, 1st–4th, and above 4th, with LAS scores as follows: below_1st = 0–33.74, 1st-4th = 33.75–44.95, and above_4th = 44.96–100. The analysis revealed a significant difference in survival among these three groups, with recipients in the first quartile having the highest 5-year survival rate and those in the lowest quartile having the poorest (p < 0.0001) (Figure 5). When further stratified into allocation score tiers, only the top third of allocation scores demonstrated significant survival with ABO identical matching (p = 0.014); the middle and lowest tiers found no significant difference (Figure 6).

**FIGURE 7:**
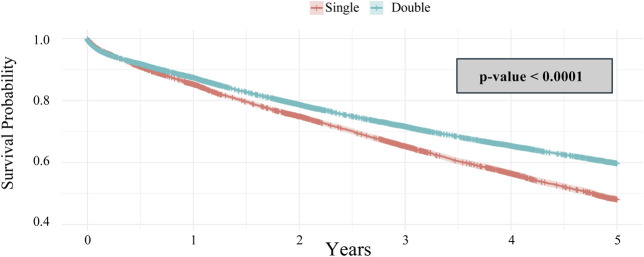
Kaplan-Meier 5-year survival analysis of lung transplant recipients categorized by single or double lung transplantation.

**FIGURE 8:**
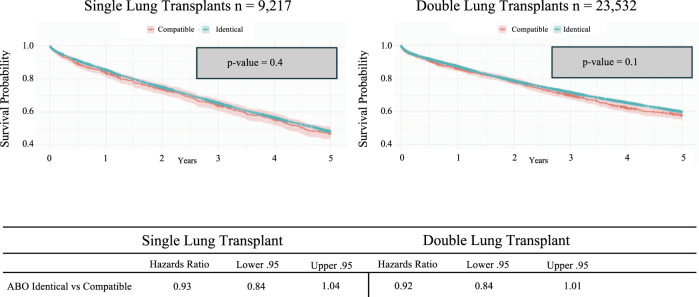
Kaplan-Meier 5-year survival analysis of lung transplant recipients categorized by ABO identical vs. compatible status receiving lung transplantation stratified by single or double lung transplantation.

Lastly, when comparing single and double lung transplantation, there was a significant difference in overall survival (Figure 7) which is consistent with previously published highlighting a survival advantage in double lung transplantation [25, 26]. However, when stratified by ABO compatibility, there is no statistical difference in either the single or double lung transplantation groups, regardless of whether ABO identical or ABO compatible matching occurred (Figure 8).

## Discussion

In sum, our work utilized the UNOS database to evaluate outcomes in ABO matching in lung transplantation. Two endpoints were examined: acute rejection and overall survival. Further sub-analysis was performed comparing ABO matching within recipient diagnosis, lung allocation score breakdown, and single versus double lung transplantation. After adjusting for confounding variables, our multivariate analysis demonstrated no significant risk of acute rejection when ABO compatible donors were used compared to the ABO identical group.

When comparing all lung transplants across the board, during the study period, our findings suggest improved long-term survival with ABO identical lung transplants. However, it was found that ABO compatible donors were significantly more likely to undergo a single lung transplant than a double lung transplant. A double lung transplant is widely known to have improved long-term survival compared to a single lung transplant [25]. When the survival data were analyzed after stratifying for transplant type, double versus single, no significant long-term survival difference was found between ABO compatible and ABO identical lung transplant recipients.

We evaluated the long-term survival of ABO compatible versus ABO identical lung transplantation based on recipient diagnosis: obstructive, restrictive, cystic fibrosis, and a fourth category, which included less ubiquitous diagnoses such as Kartagener’s syndrome, common variable immune deficiency, or associated diagnoses such as pulmonary hypertension. We found no significant difference in overall survival in all diagnostic categories except for obstructive disease. Obstructive lung disease is the only diagnosis found to be associated with better survival with ABO-identical transplantation. The biological basis for decreased survival with ABO compatible remains unclear and warrants further investigation considering our findings reflect those of an observational study with retrospective analysis of the database. Based on this, it can be inferred that for other disease categories, ABO-compatible transplantation is a viable option with favorable long-term outcomes.

We then stratified the data by allocation score, dividing it into three parts: the lowest, middle, and top tiers. It was found that only the top tier, with the highest allocation scores, showed statistically significant differences between ABO-identical and ABO-compatible transplantation in terms of overall survival. All other allocation scores showed no significant differences in survival; therefore, an ABO-compatible transplant is a reasonable option.

Chronic rejection is a major driver of reduced long-term survival after lung transplant [27, 28]. Given that immunogenicity drives risk for rejection and consequent mortality, the primary outcome of acute rejection is a useful proxy for understanding the role of ABO-compatible and ABO-identical blood typing. Fewer HLA mismatches, particularly class I, have been shown to significantly decrease the rate of chronic rejection and the development of bronchiolitis obliterans, thus influencing survival [29]. This effect has been demonstrated in the lab as well as in humans. Donor-specific anti-HLA antibodies appear to correlate with elevated defensin levels, which promote sustained inflammation and epithelial proliferation, leading to chronic rejection [30]. Class II HLA mismatching predisposes the recipient to acute rejection [29, 31]. HLA matching chronic rejection is further demonstrated in areas of the world where lobar lung transplantation is performed. Living-donor lobar lung transplant recipients appear to have immunological advantages, such as a reduced incidence and delayed onset of chronic rejection, as well as fewer *de novo* donor-specific antibodies, which may translate to improved outcomes [32]. The immunological benefits are potentially owing to HLA similarities due to blood-related donors [32]. The alloimmune response after transplant clearly plays an important role in the development of rejection and therefore significantly affects graft function both in the short and long term. Our primary analyses were limited to acute rejection events captured in the registry and all-cause long-term survival, and we were not able to assess HLA mismatch burden or clinician-diagnosed chronic rejection. An area for future study will be to test the effect of HLA mismatching in ABO-identical and compatible transplants on long-term survival in datasets that include detailed HLA typing endpoints.

We find that ABO compatible lung transplantation of both single and double lungs does not increase the risk of acute rejection. Given that single lung transplant recipients may begin at an immunological disadvantage, our findings may further support the usage of ABO compatible lung allocation as opposed to strictly ABO identical matching. Additional research is needed to examine the development of chronic rejection and its effect on long-term survival in the context of ABO compatible lung transplantation. Going one step further, ABO compatibility in lung transplantation remains in an evolutionary phase, as successful ABO-incompatible living-donor lobar lung transplantation has been reported [33].

While our findings indicate that ABO compatibility does not significantly influence acute rejection rates, it is associated with differences in survival outcomes among patients receiving a lung transplant for obstructive lung disease and for patients in the highest quartile of lung allocation scores. However, this effect was modest. This may be due to residual confounding in patients with obstructive disease and high LAS, or differential biological vulnerability to immune-mediated injury in high-risk phenotypes, such as due to passenger lymphocyte syndrome [34]. When considering factors for matching recipients and donors, results from this study underscore the importance of considering disease-specific and allocation score factors when evaluating the need for ABO identical matching. Based on our analysis, identical matching is not required for most lung transplant cases, which could shorten a patient’s waitlist time and increase the number of donor lungs available for transplantation [6].

The advent of *ex vivo* lung perfusion (EVLP) has shown tremendous promise in the field of lung transplantation, particularly in evaluating marginally acceptable organs for pre-treatment and improving organ quality [35]. An area of future direction related to the topic of this article is the possibility of converting a type A organ to type O, thereby creating a universal donor through enzymatic pre-treatment of donor lungs using EVLP [26, 35–37]. More research is needed with this novel technology, which could markedly increase ABO-compatible organs to broaden the donor pool.

These results should be evaluated in consideration of several limitations. At the same time, adjusted models accounted for several confounders, such as ABO status, recipient age, gender, medical condition, and disease classification, as an observational study can still introduce bias due to confounding. One limitation is that CLAD is a major determinant of late mortality after lung transplantation. However, CLAD is not available in a standardized form in the UNOS dataset we analyzed [38]. We therefore used acute rejection and 5-year survival as complementary endpoints. Another limitation is that our acute rejection endpoint is not time-to-event and may be affected by early death, because time-to-first acute rejection was not available in a consistent timestamped manner in our dataset. In addition, a further limitation of this study is its retrospective design, which spans from 2005 to 2023. Although the analysis spanning almost two decades may be considered a strength, variations in documentation and technology could inherently introduce several potential biases in the data set, such as evolving diagnostic and reporting standards, advancements in transplant care, and LAS implementation since its first implementation in May of 2005, in alignment with this study’s starting point. Since the conclusion of our study period, the LAS scoring system has been replaced by continuous allocation scoring; therefore, the findings may not be directly applicable to today’s lung transplant patients. Following the implementation of continuous distribution for lung allocation in the United States, we observed a significant increase in ABO-compatible transplants–doubling from 14% to 28% of annual lung transplants. The principle from our study would still be relevant, that while we believe there is a role for ABO compatible lung matching, the sickest patients may benefit from ABO identical organs.

These findings underscore the importance of considering both disease-specific factors and allocation scoring when assessing the potential benefits of ABO-matching. While identical matching may be modestly associated with a survival advantage in specific high-risk subgroups, our results support the broader use of ABO-compatible organs, which could reduce waitlist times and expand access to transplantation without increasing the risk of acute rejection while also selecting for the best candidates for compatible matching.

## Conclusion

ABO compatible lung transplants can safely be performed as there is no significant difference in acute rejection, provides a larger pool of donors, and increases access to lung transplantation. Caution needs to be taken with specific subsets of recipients, such as those in the top LAS quartile, due to their higher 5-year survival with ABO identical matching.

## Data Availability

The original contributions presented in the study are included in the article/supplementary material, further inquiries can be directed to the corresponding author.

## References

[1] Singh TP Hsich E Cherikh WS Perch M Hayes D Jr Lewis A The international thoracic organ transplant registry of the international society for heart and lung transplantation: 2025 annual report of heart and lung transplantation. J Heart Lung Transpl (2025) 44:1857–73. 10.1016/j.healun.2025.04.014 40300677

[2] ISHLT. ISHLT_Fast-Facts.pdf. Addison, Texas: International Society for Heart and Lung Transplantation (2025). Available online at: https://www.ishlt.org/docs/default-source/default-document-library/ishlt_fast-facts.pdf? (Accessed January 5, 2026).

[3] Valapour M Lehr CJ Schladt DP Swanner K Poff K Handarova D OPTN/SRTR 2023 annual data report: lung (2023). 1600–6143. 10.1016/j.ajt.2025.01.025 PMC12334194 39947808

[4] Yang Z Bai YZ Yan Y Hachem RR Witt CA Vazquez Guillamet R Validation of a novel donor lung scoring system based on the updated lung composite allocation score (2025). 1600–6143. 10.1016/j.ajt.2024.03.032 PMC11254565 38531429

[5] OPTN. Guide_To_Calculating_Lung_Composite_Allocation_Score.pdf. OPTN Lung Committee deliberations (2022).

[6] Fakhro M Larsson H Malmsjo M Algotsson L Lindstedt S . ABO-identical matching has no superiority in long-term survival in comparison to ABO-compatible matching in lung transplantation. J Cardiothorac Surg (2019) 14(1):24. 10.1186/s13019-019-0846-6 30691526 PMC6350378

[7] Snell GI Holmes M Levvey BJ Shipp A Robertson C Westall GP Lessons and insights from ABO-incompatible lung transplantation (2013). 1600–6143. 10.1111/ajt.12185 23465218

[8] Taghavi S Jayarajan SN Furuya Y Komaroff E Shiose A Leotta E Examining ABO compatible donors in double lung transplants during the era of lung allocation score. (2014). 1552–6259. 10.1016/j.athoracsur.2014.05.037 25106683

[9] Taghavi S Jayarajan SN Furuya Y Komaroff E Shiose A Leotta E Single-lung transplantation with ABO-compatible donors results in excellent outcomes (2014). 1557–3117. 10.1016/j.healun.2014.04.006 24880825

[10] Chen-Yoshikawa TF . ABO blood type incompatible lung transplantation. J Thorac Dis (2023) 15(6):3437–42. 10.21037/jtd-23-48 37426171 PMC10323570

[11] Salerno CT Burdine J Perry EH Kshettry VR Hertz MI Bolman RMI . Donor-derived antibodies and hemolysis after abo-compatible but nonidentical heart-lung and lung transplantation1. Transplantation (1998) 65(2):261–4. 10.1097/00007890-199801270-00021 9458026

[12] Pavlisko EN Neely ML Kopetskie H Hwang DM Farver CF Wallace WD Prognostic implications of and clinical risk factors for acute lung injury and organizing pneumonia after lung transplantation: data from a multicenter prospective cohort study. Am J Transpl (2022) 22(12):3002–11. 10.1111/ajt.17183 36031951 PMC9925227

[13] UNOS. New lung allocation policy in effect (2025). Available online at: https://unos.org/news/new-lung-allocation-policy-in-effect/#:∼:text=,five%20points (Accessed January 2, 2026).

[14] dplyr. A grammar of data manipulation (2025).

[15] Subirana ISH Vila J . Building bivariate tables: the compare Groups package for R. J Stat Softw (2014). 10.18637/jss.v057.i12

[16] Kassambara AKM Biecek P Pabian S . Survminer: drawing survival curves using “ggplot2.”. (2024). Available online at: https://cran.r-project.org/web/packages/survminer/index.html (Accessed December 20, 2025).

[17] Create elegant data visualisations using the grammar of graphics. (2025). Available online at: https://ggplot2.tidyverse.org/ (Accessed December 21, 2025).

[18] Wickham H Averick M Bryan J Chang W McGowan LDA François R Welcome to the tidyverse. J Open Source Softw (2019). 10.21105/joss.01686

[19] Read excel files. (2025). Available online at: https://readxl.tidyverse.org/ (Accessed December 30, 2025).

[20] Storry JR Olsson ML . The ABO blood group system revisited: a review and update (2009). 0894–203X. 19927620

[21] Usmani A Morris GP Murphey C . The increasing need for ABO blood group genotyping and quality assurance implications for laboratory implementation (2024). p. 1879–166. 10.1016/j.humimm.2024.110766 38402098

[22] Chen-Yoshikawa TF . ABO blood type incompatible lung transplantation (2023). 2072–1439. 10.21037/jtd-23-48 PMC10323570 37426171

[23] Pierson RN 3rd Loyd JE Goodwin A Majors D Dummer JS Mohacsi P Successful management of an ABO-mismatched lung allograft using antigen-specific immunoadsorption, complement inhibition, and immunomodulatory therapy. Addison, Texas: International Society for Heart and Lung Transplantation (2002). 79–84. 10.1097/00007890-200207150-00014 12134103

[24] Banner NR Rose ML Cummins D De Silva M Pottle A Lyster H Management of an ABO-Incompatible lung transplant. Am J Transplant (2004) 4(7):1192–6. 10.1111/j.1600-6143.2004.00438.x 15196081

[25] Subramanian MP Meyers BF . Bilateral versus single lung transplantation: are two lungs better than one? 2018 (2025). 2072–1439. 10.21037/jtd.2018.06.56 PMC6106016 30174911

[26] Weingarten N Mehta AC Budev M Ahmad U Yun J McCurry K Single vs double lung transplantation in older adults: a propensity-matched analysis (2025). p. 1931–3543. 10.1016/j.chest.2024.08.044 PMC11867891 39244083

[27] Bos S Vos R Van Raemdonck DE Verleden GM . Survival in adult lung transplantation: where are we in 2020? (2020). 1531–7013. 10.1097/MOT.0000000000000753 32332197

[28] Van Herck A Verleden SE Vanaudenaerde BM Verleden GM Vos R . Prevention of chronic rejection after lung transplantation (2017). 2072–1439. 10.21037/jtd.2017.11.85 PMC5756981 29312757

[29] Peltz M Edwards LB Jessen ME Torres F Meyer DM HLA mismatches influence lung transplant recipient survival, bronchiolitis obliterans and rejection: implications for donor lung allocation (2011). 1557–3117. 10.1016/j.healun.2010.10.005 21145259

[30] Saini D Angaswamy N Tiriveedhi V Fukami N Fukami N Ramachandran S Hachem R Synergistic effect of antibodies to human leukocyte antigens and defensins in pathogenesis of bronchiolitis obliterans syndrome after human lung transplantation (2010). 1557–3117. 10.1016/j.healun.2010.05.036 PMC2991612 20691611

[31] Renaud-Picard B Koutsokera A Cabanero M Martinu T . Acute rejection in the modern lung transplant era. Semin Respir Crit Care Med (2021) 42(03):411–27. 10.1055/s-0041-1729542 34030203

[32] Nakajima D Date H . Living-donor lobar lung transplantation. J Thorac Dis (2021) 13(11):6594–601. 10.21037/jtd-2021-07 34992838 PMC8662478

[33] Nakajima D Yuasa I Kayawake H Tanaka S Yamada Y Yutaka Y The first successful case of ABO-incompatible living-donor lobar lung transplantation following desensitization therapy (2023). p. 1600–6143. 10.1016/j.ajt.2023.04.031 37149042

[34] Kohl MM Schwarz S Jaksch P Muraközy G Kurz M Schönbacher M High rate of passenger lymphocyte syndrome after ABO minor incompatible lung transplantation. Am J Respir Crit Care Med (2024) 209(8):995–1000. 10.1164/rccm.202306-1107OC 38078854 PMC11531209

[35] Wang AA-O Ribeiro RA-O Ali AA-O *Ex vivo* enzymatic treatment converts blood type A donor lungs into universal blood type lungs. 2022 (2023). p. 1946–6242. 10.1126/scitranslmed.abm7190 35171649

[36] Noda K Furukawa M Chan EG Sanchez PG . Expanding donor options for lung transplant: extended criteria, donation after circulatory death, ABO incompatibility, and evolution of *Ex Vivo* lung perfusion. Transplantation (2023) 107(7):1440–51. 10.1097/tp.0000000000004480 36584375

[37] Thabut G Mal H . Outcomes after lung transplantation. J Thorac Dis (2017) 9(8):2684–91. 10.21037/jtd.2017.07.85 28932576 PMC5594127

[38] Todd JL Weigt SS Neely ML Grau-Sepulveda MV Mason K Sever ML Prognosis and risks for probable chronic lung allograft dysfunction: a prospective multicenter study. Am J Respir Crit Care Med (2025) 211(2):239–47. 10.1164/rccm.202403-0568OC 39470452 PMC11812547

